# Sense of Purpose in Caregiving: The Buffering Effect on Caregiver Burden

**DOI:** 10.1093/geroni/igaf122.3323

**Published:** 2025-12-31

**Authors:** Lindsay Wilkinson, Bethany Smith, Jong Ko

**Affiliations:** University of Nebraska Omaha, Omaha, Nebraska, United States; KEN Consulting, Inc., College Park, Maryland, United States; Johns Hopkins University, Baltimore, Maryland, United States

## Abstract

While research has predominately focused on the negative consequences of caregiving for health and well-being, less attention has been paid to the positive outcomes of caregiving. One such benefit may be the sense of purpose individuals obtain from their caregiver role. Drawing on the stress process model, this research asks two main questions: Do caregiver burden and hours spent caregiving contribute to lower perceived quality of life? If yes, does sense of purpose derived from caregiving play a moderating role in these associations? This study utilizes a sample of 53 informal caregivers to patients with chronic illnesses recruited through electronic health records. The average age of respondents was 57 years (range: 23-75), with the majority of respondents providing care to a spouse/partner (58%), followed by an aging parent (32%). Close to one-third of respondents (32%) “nearly always” felt that their life had more of a purpose since they began providing care, compared to 38% of respondents who “never” or “rarely” felt this way. Findings from linear regression models adjusting for demographic characteristics show that higher caregiver burden was significantly associated with lower quality of life (p < 0.001); however, the impact of caregiver burden was lessened among respondents who found purpose in caregiving (p < 0.01). No association was found for hours spent caregiving. This study demonstrates the importance of sense of purpose as a resource for informal caregivers, particularly those experiencing high levels of perceived burden, and could help lead to targeted interventions aimed at improving caregiver quality of life.

